# Factors Affecting the Perceived Usability of the COVID-19 Contact-Tracing Application “Thai Chana” during the Early COVID-19 Omicron Period

**DOI:** 10.3390/ijerph19074383

**Published:** 2022-04-06

**Authors:** Thanatorn Chuenyindee, Ardvin Kester S. Ong, Yogi Tri Prasetyo, Satria Fadil Persada, Reny Nadlifatin, Thaninrat Sittiwatethanasiri

**Affiliations:** 1 School of Industrial Engineering and Engineering Management, Mapúa University, 658 Muralla Street, Intramuros, Manila 1002, Philippines; thanatorn_chu@rtaf.mi.th (T.C.); aksong@mapua.edu.ph (A.K.S.O.); 2 School of Graduate Studies, Mapúa University, 658 Muralla Street, Intramuros, Manila 1002, Philippines; 3 Department of Industrial Engineering and Aviation Management, Navaminda Kasatriyadhiraj Royal Air Force Academy, Bangkok 10220, Thailand; flook46@gmail.com; 4 Entrepreneurship Department, BINUS Business School Undergraduate Program, Bina Nusantara University, Malang 65154, Indonesia; satria.fadil@binus.ac.id; 5 Department of Information Systems, Institut Teknologi Sepuluh Nopember, Kampus ITS Sukolilo, Surabaya 60111, Indonesia; reny@its.ac.id

**Keywords:** COVID-19, perceived usability, protection motivation theory, technology acceptance model, system usability scale

## Abstract

Thai Chana is one of the mobile applications for COVID-19 disease-control tracking, especially among the Thais. The purpose of this study was to determine factors affecting the perceived usability of Thai Chana by integrating protection motivation theory, the extended technology acceptance model, and the system usability scale. In all, 800 Thais participated and filled an online questionnaire with 56 questions during the early COVID-19 omicron period (15 December 2021 to 14 January 2022). Structural equation modeling (SEM) showed that the understanding of COVID-19 has significant effects on perceived severity and perceived vulnerability, which subsequently leads to perceived usefulness. In addition, perceived usefulness and perceived ease of use have significant direct effects on attitude, which subsequently leads to the intention to use, actual use, and perceived usability. This study is one of the first studies that have analyzed the mobile application for COVID-19 disease-control tracking. The significant and substantial findings can be used for a theoretical foundation, particularly in designing a new mobile application for disease-control tracking worldwide. Finally, protection motivation theory, the extended technology acceptance model, and the system usability scale can be used for evaluating other disease-control tracking mobile applications worldwide.

## 1. Introduction

The COVID-19 pandemic is one of the worst disasters in the world. As of 9 March 2022, there were 448,313,293 confirmed cases, with 6,011,482 deaths, despite the fact that a total of 10,704,043,684 vaccine doses have been administered [1]. Healthcare workers, government, and academicians are continuously involved in COVID-19 mitigation, aiming to minimize the fatalities, including the spread of the virus, while also maximizing the technology in the COVID-19 context even in mobile applications. In 2021, there were several mobile applications related to COVID-19 in use [2,3] and one of those applications was Thai Chana.

**Figure 1:**
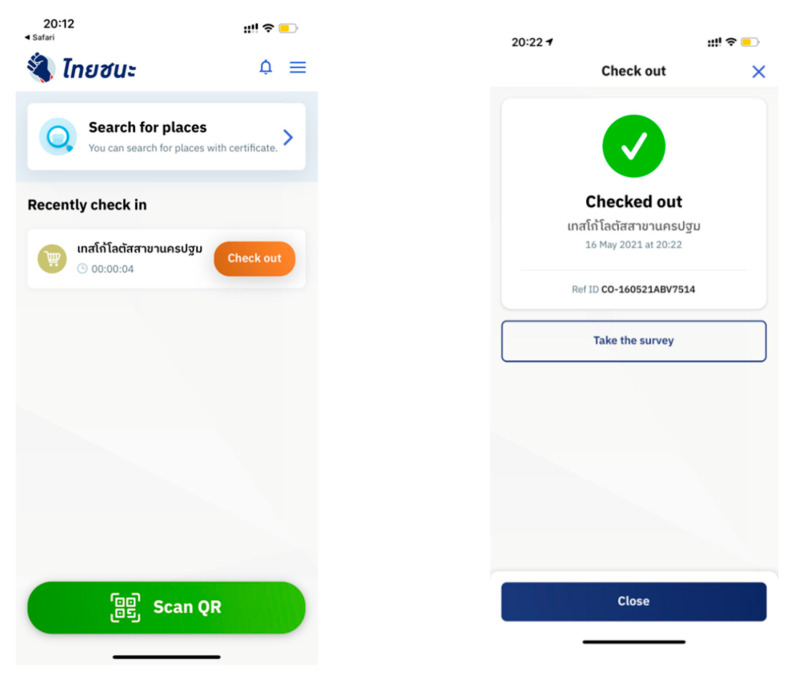
Thai Chana in iOS.

Thai Chana is an important mobile application in Thailand, developed by the government during the COVID-19 pandemic (Figure 1). It is a government-based mobile application that was created for facilitating disease-control tracking for preventing the spread of COVID-19. Thai Chana works by obtaining the user′s data through a QR code or a printed form. All Thais are required to scan the QR code or fill out the printed form every time they enter any place. If they visit a place at the same time as a COVID-19 suspect, the government will notify and call all the people who possibly came in contact with the suspect. Then, they will have to go through the COVID-19 screening test and will be forced to be quarantined. However, despite the mitigation steps taken and strategies created by the government through the mobile application, the capital of Thailand, Bangkok, is still considered one of the most highly infected areas [4]. Viwattanakulvanid [4] stated how the country is placed the second highest among Asian countries, following China. It can be seen that promoting its usage is highly important to help mitigate and reduce the infection rate in the country.

Thai Chana is a requirement for the people in Thailand, even tourists. Everyone who will enter or visit an area is required to use this mobile application. Rumyakul [5] indicated that Thai Chana is the main mobile application that should be used throughout the country. With the availability of this mobile application, Issac et al. [6] indicated how it helped the government strategize ways to mitigate the COVID-19 virus spread. In addition, Viwattanakulvanid [4] explained how Thailand was able to conduct laboratory testing, communicate risk, manage and control cases, prepare facilities, and provide medical supplies and even healthcare staff. With its implementation since the early stage of the COVID-19 spread, no studies have been conducted regarding its usability. Since Thai Chana is a new mobile application for health risk reduction, there are two important theories that can be integrated to analyze the Thais’ behavior toward Thai Chana: protection motivation theory (PMT) and the technology acceptance model (TAM).

PMT has been used to bring understanding to people fearful of the pandemic. It has been expanded further to diminish the threat and the disease. This model has turned the behavior of people in terms of cognitive perspective to protect themselves [7]. In Iran, Rad et al. [8] showed that the identification of processes has increased the defensive health behaviors of people. Similarly, in the Philippines, Prasetyo et al. [9] showed that PMT can be used in the context of the COVID-19 pandemic. Another study has revealed that public health government by the World Health Organization (WHO) is designed toward the perceived severity of the Corona virus and the health behaviors to decrease the spread of the virus [10]. In the context of the use of technology in disaster mitigation, this theory can be combined with the technology acceptance model (TAM).

TAM is one of the sophisticated models that can help people to accept and exploit new technologies. This model emphasizes the factors influencing the intention to use brand-new technologies from end to end [11,12,13]. Perceived usefulness, perceived ease of use, the attitude toward using, and the intention to use were used on Indonesian users of the COVID-19 website. It was found that all of the hypotheses and studies on each hypothesis were significantly acceptable [14]. TAM can be extended by adding perceived usability. Perceived usability is a usability evaluation through a questionnaire to estimate the usability of a product or a platform. It is also widely adopted by engineers and academicians [15], particularly in the context of human–computer interaction [16]. The system usability scale is one of the most widely used methods in measuring usability.

Previously, there have been several studies related to the usability of medical mobile applications. For patients with rheumatic diseases, Kristjansdottir et al. [17] analyzed the usability of the mobile application among 12 rheumatic patients and they found that the satisfaction point was 86.3/100. Islam et al. [18] explored the usability issues of the mHealth application, and the study showed that the services and functions were acceptable. Kuhns et al. [16,17,18,19] exposed the usability of a mobile application for HIV prevention for transgender women, and they found the satisfaction score of the application to be 4.59/5. Moreover, the participants suggested enhancing application engagement by adding user experience and user interface for attracting new users [20]. This means that enforcing the use of an application does not necessarily guarantee effectiveness. There is the need for the application to meet users’ expectations and have high usability to increase users’ willingness to use it. This would lead to a positive effect in mitigating health-related risks.

Despite the availability of several studies related to the perceived usability of a mobile application, few studies have investigated the perceived effectiveness of any mobile application mainly for COVID-19 prevention [21]. In China, Zhou et al. [22] only mentioned that the developers of mobile applications for COVID-19 need to consider the important information since the users can be overwhelmed if they received too much information. Similarly, Chidambaram et al. [23] investigated 82 applications related to COVID-19 without a strong theoretical foundation. Moreover, at present, there is no local study in Thailand that has explored further one of the most important mobile applications in Thailand, Thai Chana. Thus, it is important to explore the acceptance of Thai Chana among the Thais during the COVID-19 pandemic.

The purpose of this study was to determine factors affecting the perceived usability of Thai Chana by integrating protection motivation theory, the extended technology acceptance model, and the system usability scale. This study is one of the first studies that have analyzed the mobile application for COVID-19 disease-control tracking. Moreover, this is the first study to fully integrate PMT, the extended TAM, and the SUS. The significant and substantial findings can be used for a theoretical foundation, particularly in designing a new mobile application for disease-control tracking worldwide.

## 2. Conceptual Framework

**Figure 2:**
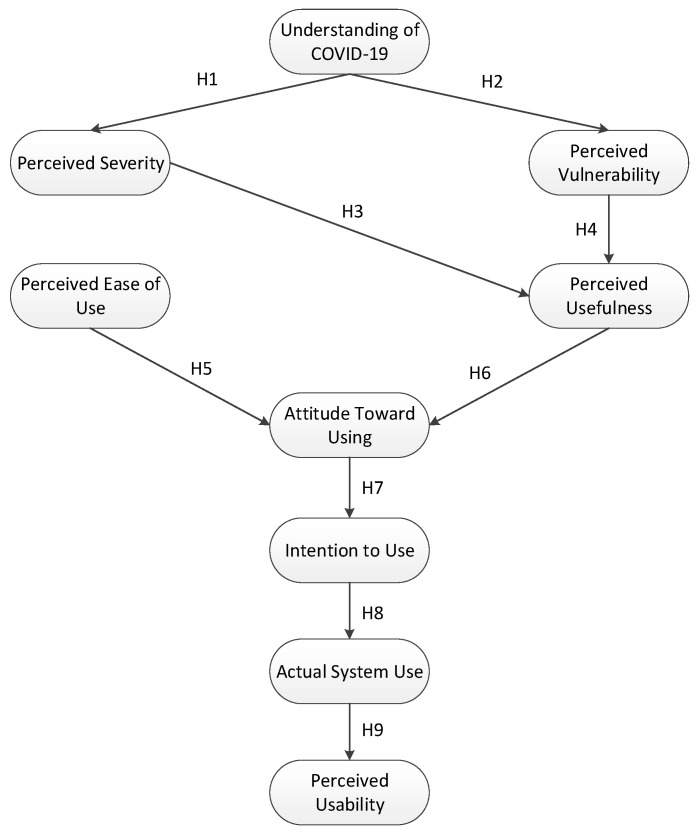
Conceptual framework.

Figure 2 represents the conceptual framework of this study. The novelty of this study is not only the investigation of factors affecting the perceived usability of a mobile application for COVID-19 contact-tracing application but also that it shows advancement of three different frameworks in one model (PMT, the extended TAM, and the SUS) in the context of the COVID-19 pandemic.

Different studies have used integrated theories to measure new applications and technologies [9,11,13,24]. Prasetyo et al. [9] considered integrating PMT and the Theory of Planned Behavior (TPB) on the perceived effectiveness of COVID-19 preventive measures. Their study highlighted how PMT factors would lead to the intention to follow mitigation of COVID-19 spread. Zhang et al. [13] explored unsafe behaviors with technology using the TPB and the TAM. The results showed that factors affecting unsafe behaviors with technology are reduced when TAM latent variables are highlighted, such as perceived ease of use and perceived usefulness. Wu and Zhang [11] extended the TAM and explained how this model has been widely used for measuring acceptance among new technologies. Other factors were suggested for consideration. Ong et al. [24] considered the integration of the TPB and PMT toward the acceptance of reusing technologies. Their study highlighted that understanding has the most significant effect on people’s acceptance. When people understand the benefits, perceived risks would be reduced, leading to high acceptance.

Studies that considered measuring usability in evaluating acceptance through the integration of the system usability scale (SUS) and the TAM have been evaluated [25]. Revythi and Tselios [25] integrated both the SUS and the TAM regarding the satisfaction of technologies. They proposed that more factors upon evaluation of technology acceptance should be explored. Al-Maatouk et al. [26] considered the task–technology fit (TTF) and TAM integration for social media adoption in the academe. The results of the study highlighted how considering the way the proper technology would fit said usage depends on its perceived usability. Lastly, PMT and UTAUT2 have been integrated to measure behavioral intentions towards using mobile health education [27]. However, UTAUT2 latent variables tend to be reduced upon evaluation of technology Thus, it is posited that integrating PMT to measure threat appraisal, the TAM for new technology acceptance evaluation, and the SUS for perceived usability could holistically measure health-related mobile applications. Presented in Table 1 is the summary of related studies that have extended and integrated several frameworks to support the hypotheses considered in this study.

Presently, a widespread epidemic era of COVID-19 originating in the Wuhan market in China has caused severe trauma worldwide. The new strain of the COVID-19 virus is spreading around the world. The World Health Organization states that the most important factor that can help to handle the COVID-19 pandemic is to understand the severity of the disease and other risk factors [23]. Some evidence has shown that elderly people with underlying medical conditions might have a higher risk of severe health problems from COVID-19 [28]. Thus, it was hypothesized that:

**H1.**  *Understanding of COVID-19 has a significant direct effect on the perceived severity*.

**H2.**  *Understanding of COVID-19 has a significant direct effect on the perceived vulnerability*.

Due to the high risk of the COVID-19 pandemic as a new disaster, many people fear being infected [6]. They have found ways to survive this pandemic by following safety protocols such as staying safe at home, following social distancing, and even working from home. Thus, in general, people really rely on the Internet service and tend to spend more time on mobile applications [29]. This new attitude toward the use of mobile applications can enhance information sharing among communities. In the context of the COVID-19 pandemic, health-related applications become crucial and Thailand’s government has developed a COVID-19 contact-tracing application named “Thai Chana.” Thus, the following were hypothesized:

**H3.**  *Perceived severity has a significant direct effect on perceived usefulness.*

**H4.**  *Perceived vulnerability has a significant direct effect on perceived usefulness*.

**H5.**  *Perceived ease of use has a significant direct effect on the attitude toward using*.

**H6.**  *Perceived usefulness has a significant direct effect on the attitude toward using*.

Thai Chana has announced the Thai government’s intention to provide COVID-19-related online data that will guide users to follow the government′s instruction. Moreover, the application is tracking people who might be at risk and notifying users around the risk area. A study by Camacho-Rivera et al. [30] revealed that 24.1% of 10,760 volunteers from America usually used their mobile phones to track and trace their location when they went to any place, the same way as the Thais use their mobile phones to trace their location using the government’s application Thai Chana. Furthermore, Pal and Vanijja [31] have revealed that the actual system is significantly affected by perceived usability. Thus, it was hypothesized that:

**H7.**  *The attitude toward using has a significant direct effect on the intention to use*.

**H8.**  *The intention to use has a significant direct effect on actual system use*.

**H9.**  *Actual system use has a significant direct effect on the perceived usability*.

## 3. Methodology

### 3.1. Respondents

This study was approved by Mapua University Research Ethics Committees and Navaminda Kasatriyadhiraj Royal Thai Air Force Academy Research Ethic Committees. Prior to data collection, all 800 Thai participants were required to fill out an online consent form that explained the objective of the study and their personal information. Hair [32] suggested that when the model considered has more than 8 latent variables, the data collection should involve more than 500 respondents for generalizability. This is justified by the study conducted by Wolf et al. [33]

All respondents (Table 2) were from Bangkok Metropolitan Region, aged between 18 and 64 years old (mean = 18–25 years old). The data collected ensured that those answering the online survey were users of Thai Chana. Those who did not use the application were not allowed to proceed with the survey. The data were collected from 15 December 2021 to 14 January 2022. Based on the descriptive statistics, 45.63% of the respondents were male and 51.88% were female. In addition, the majority were 15–24 years old (77.88%), followed by 25–34 years old (10.50%), and the rest were older. Of the respondents, 41.25% earned less than THB 15,000 (USD 454) and 43.13% earned between THB 15,000 and 30,000 (USD 454–909.85). Lastly, 60.50% were enrolled with a health insurance company and 39.50% were not.

### 3.2. Questionnaire

Table 3 represents the construct and measurement items in the study. Due to the COVID-19 pandemic, the questionnaire was distributed online by using a convenience sampling method. Information related to understanding of COVID-19 (U), perceived vulnerability (PV), perceived severity (PS), perceived ease of use (PEU), perceived usefulness (PU), attitude toward using (A), intention to use (IU), actual system use (AU), and even perceived usability (PU) was gathered to determine the contributing factors toward the perceived usefulness among the Thais. Furthermore, a 5-point Likert scale was used to measure the response.

### 3.3. Structural Equation Modeling

Structural equation modeling (SEM) was used to measure the causal relationships between latent variable constructs. It was run by using AMOS 24. Following some previous studies [39,40,41,42,43], SEM could be used to assess human behavior when it comes to acceptance, intention, and usage. Behkami and Daim [44] used SEM to explore technology adoption among patient-centered medical homes. Ducey and Coovert [45] considered SEM in evaluating tablet computer use, considering the technology acceptance model. Pendergrass and Ranganthan [46] used SEM in determining factors affecting electronic health information exchange in the United States. Thus, this study considered using SEM in determining factors affecting the perceived usability of Thai Chana as a COVID-19 contact-tracing application.

## 4. Results

**Figure 3:**
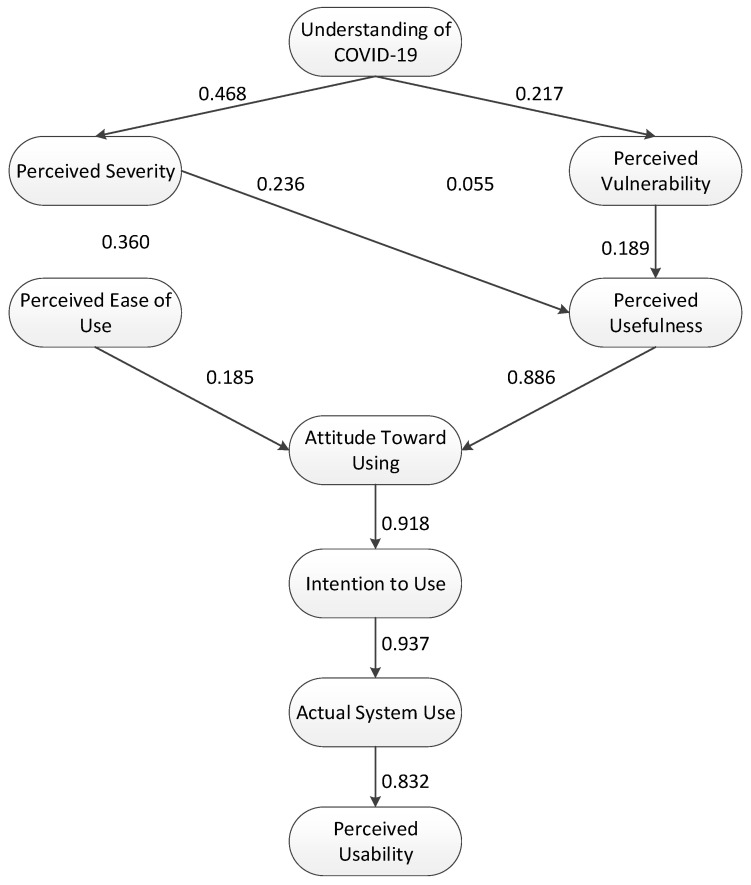
The final SEM for evaluating the perceived usability of the Thai Chana mobile application.

Table 4 represents the model fit, indicating that all parameters are within the threshold. In addition, Figure 3 presents the final SEM, according to which, it can be stated that the model is acceptable [24,32,47].

Table 5 presents the reliability and validity of the constructs. Based on the results, there is internal reliability and validity of the constructs due to Cronbach′s α and CR having values greater than 0.7 [32] and AVE having a value greater than 0.4 [48]. Moreover, Table 6 presents the causal relationship of the model. Following the suggestion of different studies [16,24], a *p*-value of 0.05 was considered the threshold for the presence of a significant relationship.

## 5. Discussion

As per the results, IU on AU showed the highest significant effect (β: 0.937; *p* = 0.001), followed by A on IU (β: 0.918; *p* = 0.001). The indicators show that the Thais would be willing, promote, and continue to use Thai Chana. Individuals will follow the protocol implemented and announcements by the government. This was preceded by having the notion that Thai Chana will be beneficial, endow a feeling of safety and security, lessen stress, and promote health. These will promote AU of the contact-tracing application. Similar to the results of Velicia-Martin et al. [38], users with a higher understanding of educational attainment do believe that the COVID-19 contact-tracing application is extremely useful. Moreover, the security concern was found to be substantial in terms of how private the information is once gathered. Individuals from the Philippines in the study by Ong et al. [34] indicated that attitude has a significant indirect effect on an individual’s actual action, which supports the findings of this study (β: 0.861; *p* = 0.001). Kurniasih et al. [14] and Walrave et al. [49] indicated that IU has a significant effect on AU. Individuals from Belgium likely have a positive innovativeness application attitude to the intention to use technology due to PU.

PU was seen to have a significant direct effect on A (β: 0.886; *p* = 0.001). Indicators highlighted safety, responsiveness, usefulness, preparedness, and awareness upon using the application. Subsequently, PEU was seen to have a low significant direct effect on A (β: 0.185; *p* = 0.001). The application is clear, understandable, easy to use, and accurate information is received upon using the application. Both PU and PEU have been investigated to have synergistic effects that lead to AU with the TAM [50]. Similarly, Camacho-Rivera et al. [30] found that 21.8% of US citizens use COVID-19 trackers due to usefulness and PEU. Based on the results of Zeng and Li [51], PU will lead to a positive indirect effect on the intention of an individual. Moreover, the PEU of technology would have an effect on the AU of technology [52].

It was also seen that AU has a highly significant direct effect on SUS (β: 0.835; *p* = 0.001). Thai individuals have the intention to install, use, read, and follow Thai Chana and are satisfied with it. The promotion by the government and the implied advantages of the contact-tracing application were seen to be beneficial among people. People have the perception that on using the application, they will remain safe and healthy. Pal and Vanijja [31] deferred that AU has a significant effect on the SUS. Russ and Salem [53] explained how end-users are highly significant in determining the usability of technology. It was deduced that the design and the technology flow should be evaluated by end-users to highlight how people will adapt to using the application. Borsci et al. [54] highlighted how usability could be a starting point to evaluate technology and to further enhance a system for efficient and actual usage among individuals. Moreover, the study of Russ and Salem [53] also indicated how measurement scales should be implemented to effectively measure the usability of a system, wherein this study adapted the SUS. This will enable the development of technologies, such as Thai Chana, that are accepted by users.

U was found to also have a significant direct effect on PS (β: 0.468; *p* = 0.001) and PU (β: 0.217; *p* = 0.001) that could be compared with the results of Ong et al. [34]. Their study explained how understanding would lead to a positive attitude and intention among people if the threat is present, especially when health and safety is the subject. People would have the intention if they perceive high levels of vulnerability and severity of impact [55]. Based on the indicators, it was seen that Thai people generally have knowledge and understanding of COVID-19 and its transmission, protocols, hospitalization, and vaccination. The more high the people perceive the threat to be, the more likely it is that they will not have a positive actual use and acceptance of technology [24].

PS (β: 0.236; *p* = 0.001) and PV (β: 0.189; *p* = 0.001) were seen to have a significant direct effect on PU. Vulnerability among family and friends and of self, area, and country were key indicators for PV, while serious health severity, death, expense, exposure, and seriousness of disease were key indicators for PS. Walrave et al. [37] explained how the usage of an application is preceded by a significant positive influence based on health confidence. This indicates that if people have negative health implications, people would tend to mitigate the impact as much as possible. Supporting this, Lewis [36] deduced that the use of the COVID-19 contact-tracing application would reduce the spread by prevention. Thus, the perception of health risk would greatly influence people’s perception of usefulness of the application Thai Chana.

### 5.1. Theoretical and Practical Contributions

The integration of PMT, the extended TAM, and the SUS showed how technology could holistically measure intention and perceived usability. With the integration, many more factors were considered compared to individual frameworks. In addition, the integrated framework may be considered to measure other tracing applications, technology, and software/application worldwide. People will have a positive attitude toward the actual use of the application when they understand COVID-19 and know the severity, vulnerability, and health impact of the virus. The government may highlight this to promote the use of Thai Chana. This will help in the monitoring and continuous use of the application to reduce the exposure to and risk of COVID-19 among people in Thailand. The government may also advertise the advantage of tracing applications to promote mitigation and preparedness.

From the findings of the study, it would be best for developers to highlight the understanding of the risk that the COVID-19 virus can affect people’s lives. This will lead to them perceiving vulnerability, which would have an indirect relationship with the perceived usability of the Thai Chana COVID-19 contact-tracing application. In addition, the understanding of COVID-19 may enhance the perceived severity of the COVID-19 spread since all countries are suffering from the effect of the pandemic. All factors would lead to a positive influence of the perceived usefulness. Moreover, the perceived ease-of-use factor should be considered by developers, which indicates that the Thai Chana application is easy to navigate and at the same time provides timely updates and information to people. This will affect the attitude and, thus, the intention to continuously patronize the mobile application. Overall, when people’s mindset and expectations are met in using a health-related mobile application, a positive influence on perceived usability will be seen [56]. This is applicable for both commercial and government-implemented software applications. Successful implementation would be based on the actual system usage and perceived usability.

### 5.2. Limitations

Despite the significant findings presented in this study, there are some limitations of the study. First, the data were gathered online through a self-administered questionnaire. Thus, the factors considered were the ones incorporated in this study. Other factors may be considered if an interview is conducted. Second, this study was not able to collect data from people representing all areas of Thailand. People from rural areas may have other considerations for the usability of Thai Chana. Therefore, people without Internet access may be considered. Lastly, some Thai respondents, especially the older generation, only used printed forms of Thai Chana. This study measured only the perceived effectiveness rather than the actual effectiveness. Future research could integrate the perceived effectiveness with the number of active cases of COVID-19.

## 6. Conclusions

Thai Chana is one of the mobile applications for COVID-19 disease-control tracking, especially among the Thais. The purpose of this study was to determine factors affecting the perceived usability of Thai Chana by integrating PMT, the extended TAM, and the SUS. In all, 800 Thais participated and filled an online questionnaire with 56 questions. SEM showed that an understanding of COVID-19 had a significant effect on perceived severity, which subsequently led to perceived ease of use and perceived usefulness. Perceived ease of use and perceived usefulness were found to have significant effects on the attitude toward using, which subsequently led to the intention to use and actual system use. Finally, actual system use was found to have a significant effect on perceived usability. If people understand the severity and the advantage of using the application, they are more likely to consider it. This study is the first study to analyze the mobile application for COVID-19 disease-control tracking in Thailand. Moreover, this is the first study to fully integrate PMT, the extended TAM, and the SUS. The significant and substantial findings can be used as a theoretical foundation, particularly in designing a new mobile application for disease-control tracking worldwide [57,58,59,60,61,62].

## Figures and Tables

**Table 1:** Summary of related studies.

Author	Year	Theory	Findings
Prasetyo et al. [9]	2020	Integrated PMT and TPB	Measured the perceived effectiveness of COVID-19 protocols. PMT factors greatly influenced the positive results.
Wu and Zhang [11]	2014	Extended TAM	Showed how TAM has been widely used for measuring acceptance among new technologies. Other factors were suggested to be considered.
Zhang et al. [13]	2020	Integrated TAM and TPB	Showed how perceived ease of use and perceived usefulness would reduce unsafe behavior in using technology.
Ong et al. [24]	2021	Integrated PMT and TPB	Indicated that understanding the benefits would lead to a reduction in perceived risk when considering the acceptance of technology.
Revythi and Tselios [25]	2019	Integrated SUS and TAM	Advised that consideration of more factors will holistically measure technology acceptance.
Al-Maatouk et al. [26]	2020	Integrated TAM and TTF	Indicated that proper consideration of technology is needed depending on the fit regarding its applicability.
Yu et al. [27]	2021	Integrated PMT and UTAUT2	Indicated that a reduction in latent variables in UTAUT2 is usually evident when considering technology evaluation.

**Table 2:** The demographic profile of the respondents (*N* = 800).

Characteristics	Category	*N*	%
Gender	Male	365	45.63
	Female	415	51.88
	Other	20	2.50
Age	15–24	623	77.88
	25–34	84	10.50
	35–44	34	4.25
	45–54	31	3.88
	55–64	27	3.88
	More than 64	1	0.13
Monthly Salary/Allowance	THB < 15,000	330	41.25
	THB 15,000–30,000	345	43.13
	THB 30,000–45,000	64	8.00
	THB 45,000–60,000	30	3.75
	THB 60,000–75,000	12	1.50
	>THB 75,000	18	2.25
Enrolled with a Health Insurance Company?	Yes	484	60.50
	No	316	39.50

**Table 3:** Construct and measurement items.

Construct	Items	Measures	Supporting References
Understanding of COVID-19	U1	I do understand the transmission of COVID-19.	Prasetyo et al. [9]
	U2	I do understand the incubation period of COVID-19.	Li et al. [34]
	U3	I do understand the general symptoms of COVID-19.	Munzert et al. [35]
	U4	I do understand the protocol if I have the symptoms that might lead to COVID-19.	
	U5	I do understand which hospital can treat COVID-19 patients.	
	U6	I do understand when I can get the vaccine for COVID-19 from the Thai government.	
Perceived Vulnerability	PV1	I think I am vulnerable to COVID-19.	Prasetyo et al. [9]
	PV2	I think my area is vulnerable to COVID-19.	Kowalski and Black [10]
	PV3	I think there is a chance that my family will be infected by COVID-19.	
	PV4	I think my friends/colleagues are vulnerable to COVID-19.	
	PV5	I think Thailand is more vulnerable than ASEAN countries.	
Perceived Severity	PS1	I find COVID-19 is a serious disease.	Prasetyo et al. [9]
	PS2	I find COVID-19 can lead to sudden death.	
	PS3	I find COVID-19 is more severe than other diseases.	Kowalski and Black [10]
	PS4	I find COVID-19 can affect my mental health.	
	PS5	I think it is expensive to pay for the medical expenses for COVID-19.	Lewis [36]
	PS6	I think the COVID-19 outbreak will continue until the middle of 2021.	Walrave et al. [37]
	PS7	I think COVID-19 in Thailand is more severe than ASEAN countries.	
Perceived Ease of Use	PEU1	I think Thai Chana can provide information related to COVID-19 that I want.	Prasetyo et al. [9]
	PEU2	Information provided by Thai Chana is clear and understandable.	Kurniasih et al. [14]
	PEU3	I can use Thai Chana successfully every time.	
	PEU4	I believe the information provided by Thai Chana is correct.	Camacho-Rivera et al. [30]
	PEU5	It would be easy for me to become skillful at using Thai Chana.	
Perceived Usefulness	PU1	Using Thai Chana would protect me from COVID-19.	Prasetyo et al. [9]
	PU2	Using Thai Chana can enhance my health.	Kurniasih, et al. [14]
	PU3	The COVID-19 spread map can enhance my awareness and preparedness.	Camacho-Rivera et al. [30]
	PU4	Safety guidelines in Thai Chana are useful.	
	PU5	Announcements in Thai Chana are useful.	
	PU6	Hotline number in Thai Chana is responsive.	
	PU7	Using Thai Chana can save my community from COVID-19.	
Attitude Toward Using	A1	Thai Chana is beneficial for me.	Prasetyo et al. [9]
	A2	Thai Chana makes me feel safe from COVID-19.	Kurniasih et al. [14]
	A3	Thai Chana can reduce my stress due to COVID-19.	Velicia-Martín et al. [38]
	A4	Thai Chana gives the community a sense of security.	
	A5	I feel I have to use Thai Chana for the sake of my health.	
Intention to Use	IU1	I will be willing to use Thai Chana in the future.	Prasetyo et al. [9]
	IU2	I will continue to use Thai Chana in the future.	Kurniasih et al. [14]
	IU3	I will promote Thai Chana to other people in the future.	
	IU4	I will follow the announcements by the government in Thai Chana.	
	IU5	I will follow the health protocol in Thai Chana.	
Actual System Use	AU1	I intend to install Thai Chana on my device.	Prasetyo et al. [9]
	AU2	Most people in my community are using Thai Chana.	
	AU3	I feel insecure if I do not use Thai Chana.	Pal and Vanijja [31]
	AU4	I often read announcements in Thai Chana.	
	AU5	I follow the safety guidelines provided by Thai Chana.	
	AU6	I feel satisfied with Thai Chana.	
Perceived Usability	SUS1	I think I would use this system frequently.	Prasetyo et al. [9]
	SUS2	I think Thai Chana is unnecessarily complex.	Orfanou et al. [15]
	SUS3	I think Thai Chana is easy to use.	
	SUS4	I think I can operate Thai Chana by myself without technical support.	Pal and Vanijja [31]
	SUS5	I find that various functions in Thai Chana are well integrated.	
	SUS6	I think the Thai Chana system is consistent.	
	SUS7	I would imagine many people in Thailand will use Thai Chana.	
	SUS8	I think it is comfortable using Thai Chana.	
	SUS9	I feel confident using Thai Chana.	
	SUS10	I do not need to learn many things before using Thai Chana.	

**Table 4:** Model fit.

Goodness-of-Fit Measures of the SEM	Parameter Estimates	MinimumCut-Off	Recommended by
Goodness-of-Fit Index (GFI)	0.879	>0.80	Gefen et al. [47]
Adjusted Goodness-of-Fit Index (AGFI)	0.853	>0.80	Gefen et al. [47]
Root Mean Square Error of Approximation (RMSEA)	0.050	<0.07	Hair [32]
Incremental Fit Index (IFI)	0.959	>0.90	Hair [32]
Tucker Lewis Index (TLI)	0.955	>0.90	Hair [32]
Comparative Fit Index (CFI)	0.959	>0.90	Hair [32]

**Table 5:** Tests of reliability and validity.

Latent Variables	Items	Factor Loadings	Cronbach′s α	Average Variance Extracted (AVE)	Composite Reliability (CR)
**U**	U1	0.64	0.789	0.432	0.817
	U2	0.76			
	U3	0.72			
	U4	0.72			
	U5	0.58			
	U6	0.48			
**PV**	PV1	0.84	0.879	0.604	0.881
	PV2	0.75			
	PV3	0.88			
	PV4	0.84			
	PV5	0.53			
**PS**	PS1	0.75	0.843	0.445	0.846
	PS2	0.73			
	PS3	0.82			
	PS4	0.64			
	PS5	0.61			
	PS6	0.51			
	PS7	0.57			
**PEU**	PEU1	0.83	0.932	0.738	0.934
	PEU2	0.90			
	PEU3	0.79			
	PEU4	0.91			
	PEU5	0.86			
**PU**	PU1	0.87	0.962	0.783	0.962
	PU2	0.86			
	PU3	0.87			
	PU4	0.89			
	PU5	0.91			
	PU6	0.89			
	PU7	0.91			
**A**	A1	0.90	0.965	0.811	0.956
	A2	0.91			
	A3	0.91			
	A4	0.89			
	A5	0.89			
**IU**	IU1	0.89	0.964	0.808	0.955
	IU2	0.90			
	IU3	0.91			
	IU4	0.91			
	IU5	0.90			
**AU**	AU1	0.89	0.963	0.783	0.956
	AU2	0.90			
	AU3	0.85			
	AU4	0.89			
	AU5	0.88			
	AU6	0.90			
**SUS**	SUS1	0.84	0.962	0.699	0.958
	SUS2	0.66			
	SUS3	0.84			
	SUS4	0.76			
	SUS5	0.91			
	SUS6	0.92			
	SUS7	0.88			
	SUS8	0.91			
	SUS9	0.88			
	SUS10	0.73			

**Table 6:** Direct effect, indirect effect, and total effect.

No	Variables	Direct Effect	*p*-Value	Indirect Effect	*p*-Value	Total Effect	*p*-Value
1	U → PV	0.217	0.001	-	-	0.217	0.001
2	U → PS	0.468	0.001	-	-	0.468	0.001
3	U → PU	-	-	0.151	0.001	0.151	0.001
4	U → A	-	-	0.134	0.001	0.134	0.001
5	U → IU	-	-	0.123	0.001	0.123	0.001
6	U → AU	-	-	0.115	0.001	0.115	0.001
7	U → SUS	-	-	0.096	0.001	0.096	0.001
8	PEU → A	0.185	0.001	-	-	0.185	0.001
9	PEU → IU	-	-	0.170	0.001	0.170	0.001
10	PEU → AU	-	-	0.159	0.001	0.159	0.001
11	PEU → SUS	-	-	0.132	0.001	0.132	0.001
12	PV → PU	0.189	0.001	-	-	0.189	0.001
13	PV → A	-	-	0.167	0.001	0.167	0.001
14	PV → IU	-	-	0.154	0.001	0.154	0.001
15	PV → AU	-	-	0.144	0.001	0.144	0.001
16	PV → SUS	-	-	0.120	0.001	0.120	0.001
17	PS → PU	0.236	0.001	-	-	0.236	0.001
18	PS → A	-	-	0.209	0.001	0.209	0.001
19	PS → IU	-	-	0.192	0.001	0.192	0.001
20	PS → AU	-	-	0.180	0.001	0.180	0.001
21	PS → SUS	-	-	0.150	0.001	0.150	0.001
22	PU → A	0.886	0.001	-	-	0.886	0.001
23	PU → IU	-	-	0.814	0.001	0.814	0.001
24	PU → AU	-	-	0.763	0.001	0.763	0.001
25	PU → SUS	-	-	0.635	0.001	0.635	0.001
26	A → IU	0.918	0.001	-	-	0.918	0.001
27	A → AU	-	-	0.861	0.001	0.861	0.001
28	A → SUS	-	-	0.716	0.001	0.716	0.001
29	IU → AU	0.937	0.001	-	-	0.937	0.001
30	IU → SUS	-	-	0.780	0.001	0.780	0.001
31	AU → SUS	0.835	0.001	-	-	0.835	0.001

## Data Availability

The data presented in this study are available on request from the corresponding author.
